# Spatio-Temporal Cluster Detection of Dengue, Chikungunya, and Zika Viruses’ Infection in Rio de Janeiro State from 2010 to 2019

**DOI:** 10.3390/v15071496

**Published:** 2023-07-01

**Authors:** Paula Maria Pereira de Almeida, Daniel Cardoso Portela Câmara, Aline Araújo Nobre, Tania Ayllón, Mário Sérgio Ribeiro, Cristina Maria Giordano Dias, Eduardo Mesquita Peixoto, Maíra Mendonça da Rocha, Silvia Carvalho, Nildimar Alves Honório

**Affiliations:** 1 Laboratório das Interações Vírus Hospedeiros, Instituto Oswaldo Cruz, Fundação Oswaldo Cruz, Rio de Janeiro 210400-900, RJ, Brazil; dcpcamara@gmail.com; 2 Núcleo Operacional Sentinela de Mosquitos Vetores-Nosmove, Fundação Oswaldo Cruz, Rio de Janeiro 21400-900, RJ, Brazil; tayllon@ucm.es; 3 Secretaria de Estado de Saúde do Rio de Janeiro, Rio de Janeiro 20031-142, RJ, Brazil; mario_sesrj@yahoo.com.br (M.S.R.); crismgd10@gmail.com (C.M.G.D.); eduardo.mesquita.peixoto@gmail.com (E.M.P.); mairamendoncadarocha@gmail.com (M.M.d.R.); carvalho.silviacristina@gmail.com (S.C.); 4 Programa de Computação Científica, Fundação Oswaldo Cruz, Rio de Janeiro 210400-900, RJ, Brazil; aline.nobre@fiocruz.br; 5 Department of Genetics, Physiology and Microbiology, Faculty of Biological Sciences, University Complutense of Madrid, 28040 Madrid, Spain; 6 Escola Nacional de Saúde Pública, Fundação Oswaldo Cruz, Rio de Janeiro 210400-900, RJ, Brazil

**Keywords:** arbovirus infections, *Aedes aegypti*, Rio de Janeiro state, Brazil, spatio-temporal analysis

## Abstract

Dengue (DENV), chikungunya (CHIKV), and Zika (ZIKV) virus infections are widespread throughout the Rio de Janeiro state. The co-circulation of these emergent arboviruses constitutes a serious public health problem, resulting in outbreaks that can spatially and temporally overlap. Environmental conditions favor the presence, maintenance, and expansion of *Aedes aegypti*, the primary vector of these urban arboviruses. This study assessed the detection of clusters of urban arboviruses in the Rio de Janeiro state from 2010 to 2019. Notified cases of dengue, chikungunya, and Zika were grouped by year according to the onset of symptoms and their municipality of residence. The study period recorded the highest number of dengue epidemics in the state along with the simultaneous circulation of chikungunya and Zika viruses. The analyzes showed that the central municipalities of the metropolitan regions were associated with higher risk areas. Central municipalities in metropolitan regions were the first most likely clusters for dengue and Zika, and the second most likely cluster for chikungunya. Furthermore, the northwest and north regions were comprised clusters with the highest relative risk for the three arboviruses, underscoring the impact of these arboviruses in less densely populated regions of Brazil. The identification of high-risk areas over time highlights the need for effective control measures, targeted prevention and control interventions for these urban arboviral diseases.

## 1. Introduction

Historically, the Rio de Janeiro state has been susceptible to the establishment and expansion of urban arboviruses due to a complex mix of socioeconomic, environmental, and demographic factors that favor the presence of *Aedes aegypti* (Linnaeus, 1762), the main vector. Dengue virus (DENV), chikungunya virus (CHIKV), and Zika virus (ZIKV) are the three main urban arboviruses circulating in Brazil. DENV is the predominant arbovirus that has consistently caused outbreaks for over 30 years in the Rio de Janeiro state. The dengue outbreaks are likely a contributing factor to the introduction and spread of DENV to other regions in Brazil. Specifically, three of the four dengue serotypes had their presence detected for the first time in the state of Rio de Janeiro: DENV-1 in 1986, DENV-2 in 1990 and DENV-3 in 2000 [1,2,3]. Dengue has become endemic-epidemic, and since then the state of Rio de Janeiro has constantly reported fatal cases [4,5,6]. According to the Pan American Health Organization (PAHO), there has been a decrease in the notification of confirmed dengue cases, especially in South and Central America, since the onset of the COVID-19 pandemic in 2020 [7]. 

In Brazil, arthropod-borne diseases substantially influence human health and well-being. For example, arboviral infections can cause microcephaly in babies associated with ZIKV infection in pregnant women, persistent arthritis due to CHIKV infection, Guillain–Barré syndrome, and severe and hemorrhagic dengue [8,9,10]. Additionally, climate change is likely to contribute to further geographic expansion of *Aedes* (*Stegomyia*) mosquitoes [11]. The co-circulation of DENV, CHIKV, and ZIKV is favored by increased globalization and the presence of peridomestic mosquitoes, such as *Ae. aegypti* and *Ae. albopictus* (Skuse, 1894) populations which are prone to arboviral infection [12,13]. As a result, this situation constitutes a significant public health challenge and a substantial economic burden [14].

Spatio-temporal analysis studies can be used as a tool to optimize arbovirus prevention strategies aimed at identifying high-risk dengue, chikungunya, and Zika clusters in space and time [13,15]. Moreover, this technique has been applied to visualize and understand the distribution of these urban arboviruses, highlighting the formation of different patterns depending on environmental conditions across a range of diverse locations. In fact, cluster analysis can be used for the development of more effective arbovirus control programs with enhanced emergency response, especially for areas determined to be at persistent risk for dengue, chikungunya, and Zika [15,16,17,18,19,20]. This process is facilitated through stratification which involves categorizing and ranking information to establish orders of importance. It allows identifying areas where the highest number or proportion of cases occurs, outlining high-risk zones. In Brazil, health surveillance programs typically stratify areas at high risk of transmission based on historical data (particularly for dengue), persistence of cases in the locality, and population at risk. Stratification proves to be a valuable tool in identifying high-risk transmission areas and understanding the spatial distribution patterns of dengue, chikungunya, and Zika. Therefore, it helps to determine where most cases are concentrated and highlights areas with greater persistence of transmission [20,21].

In this study, we conducted a spatio-temporal analysis of notified cases of DENV, CHIKV, and ZIKV infection, evaluated how the three arboviruses correlate in time and space, and identified clusters indicating arbovirus high-risk areas in the state from 2010 to 2019 for dengue, and from 2015 to 2019 for chikungunya and Zika.

## 2. Materials and Methods

### 2.1. Study Site

**Figure 1:**
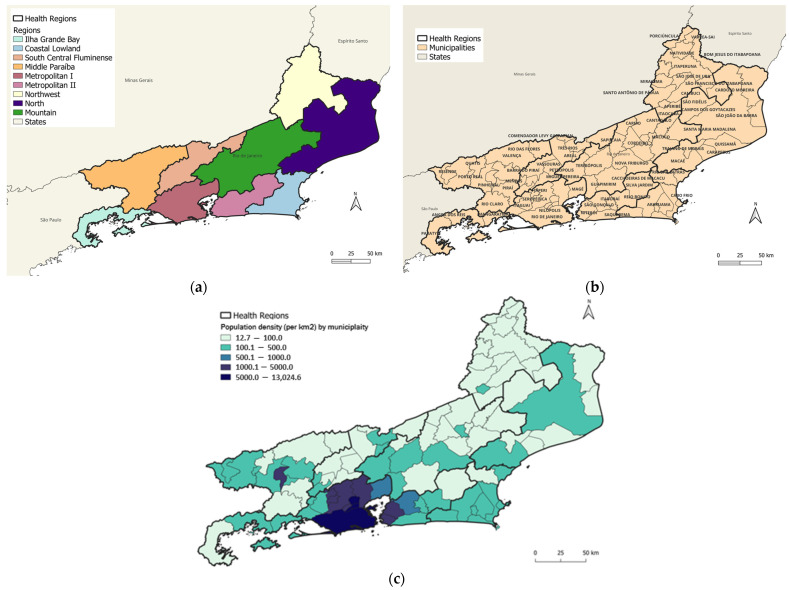
Map of Rio de Janeiro state showing (**a**) health regions; (**b**) municipalities; and (**c**) population density.

Rio de Janeiro state is located in the southeast region of Brazil, with an area of 43,750 km^2^ and an estimated population of 17,463,349. It has the second highest population density in the country, with 365 inhabitants per km^2^ [22]. The Rio de Janeiro state is divided into nine health regions aiming at organizing health actions and services in care networks [23] (Figure 1a) comprising 92 municipalities (Figure 1b) which constituted the units of analysis. The municipalities with the highest population density were concentrated in the Metropolitan I and Metropolitan II regions (Figure 1c). The state is also part of the Atlantic Forest biome, with a diverse landscape of mountains, lowlands, sandbanks, bays, lagoons, and tropical forests [22,24].

### 2.2. Study Design and Data Source

An ecological study of urban arboviruses was conducted in the Rio de Janeiro state to provide a spatio-temporal analysis of notified cases of dengue, chikungunya, and Zika to identify significant clusters from 2010 to 2019. Data on notified cases were obtained from the National System of Notifiable Diseases (SINAN) of the Rio de Janeiro state Department of Health (SES/RJ) for the period of 2010–2019. The notified cases were aggregated by the month of symptom onset to generate a time series for analysis. Data were grouped by the year of symptom onset and municipality of residence for the cluster detection. 

The population data and map shape were obtained from the Brazilian Institute of Geography and Statistics (IBGE) website [22].

### 2.3. Spatio-Temporal Analyses

Each arbovirus was analyzed separately. As chikungunya and Zika were introduced more recently, two periods were used in the analysis: from January 2010 to December 2019 for the dengue analysis, and from January 2015 to December 2019 for the chikungunya and Zika analyses.

In the spatio-temporal cluster analysis, the centroid of each municipality was extracted, and the total number of cases was aggregated for each year of the analysis for each arbovirus. The statistical scanning method proposed by Kulldorff [25] was used to identify spatio-temporal clusters, with the municipality as the spatial unit and the year as the temporal unit [25]. Using moving cylinders across space (i.e., the base of the cylinder) and time (i.e., the height of the cylinder), the scanning method identified high-risk clusters by comparing the observed number of cases to the expected number of cases inside the cylinder under a null hypothesis that the risk within the cylinder is equal to the one outside of it. A retrospective spatio-temporal analysis was performed using the Poisson probability distribution for dengue, chikungunya, and Zika [26].

To test the null hypothesis that risk within the cylinder is equal to the one outside of it, the Monte Carlo approach was used with 999 repetitions and a *p*-value of 0.05 as the threshold for statistical significance. The log-likelihood ratio (LLR) was used to test cluster formation. The higher the LLR, the greater the probability of a cluster. The detection of clusters was delimited considering 50% of the resident population of the state with a coverage radius of up to 65 km, which was determined after stratifying within each health region the minimum (3 km) and maximum (62 km) values of the average distance between two municipalities [13]. For each generated cluster, the log-likelihood ratio (LLR) was calculated, which was based on the number of cases observed inside and outside the cluster. The cluster that had the highest value for this metric was called the most likely cluster. Thus, the LLR considers the number of cases observed inside and outside the cluster, pointing to the most admissible cluster. The Relative Risk (RR) was calculated by comparing the risk in a zone in relation to the risk outside the zone. If these risks are equal, the RR is equal to 1 [26]. 

Demographic (population density) and the House Index (HI) entomological indicators (*Ae. aegypti* HI) were used to characterize the clusters in an exploratory analysis. As time scales are very different between each set of variables (population data were collected once in 2010; entomological data were collected once each year in a single week, according to the Larval Index Rapid Assay for *Ae. aegypti* (LIR*Aa*) surveillance guidelines in Brazil), we chose to focus on these guidelines for Brazil and not to proceed with other, more in-depth analyses. Softwares R version 4.2.3 (The R Foundation for Statistical Computing, Vienna, Austria), SaTScan version 10.0.1 (SaTScan^TM^, Calverton, MA, USA) and QGIS version 3.28.5 (QGIS Development Team, QGIS Association, USA) were used to perform the data analysis.

## 3. Results

### 3.1. Exploratory Analysis

**Figure 2:**
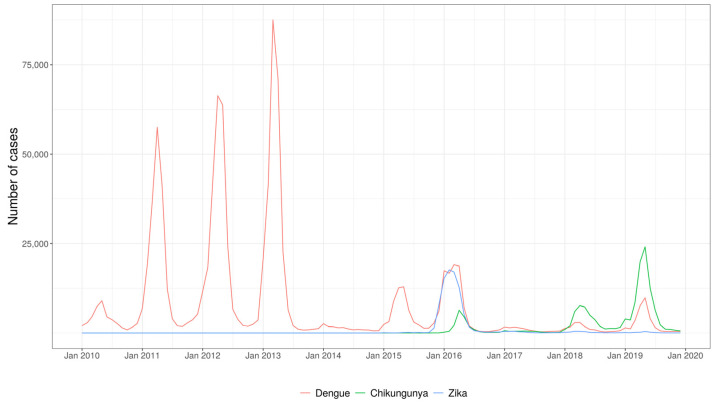
Time series of reported cases of dengue, chikungunya and Zika, from 2010 to 2019, Rio de Janeiro state.

From 2010 to 2019, a total of 1,315,182 cases of arboviruses were reported in the Rio de Janeiro state consisting of 1,049,002 (79.76%) dengue, 166,686 (12.67%) chikungunya, and 99,494 (7.57%) Zika cases. Figure 2 shows the number of noted dengue, chikungunya, and Zika cases in the state from 2010 to 2019. In the monthly curve of the historical series of cases, dengue stands out for the longest period of occurrence, with a record of five epidemic years, from 2011 to 2013 and from 2015 to 2016. The number of chikungunya cases increased in 2016 and 2018; however, further increases in cases and the associated transmission were observed in 2019. Zika stands out only in the year 2016, with high transmission. Dengue presents epidemics of greater magnitude before the introduction of chikungunya and Zika, and the year 2016 stands out with an increase in the transmission of the three arboviruses in the state.

### 3.2. Spatio-Temporal Cluster Analysis of Dengue

**Figure 3:**
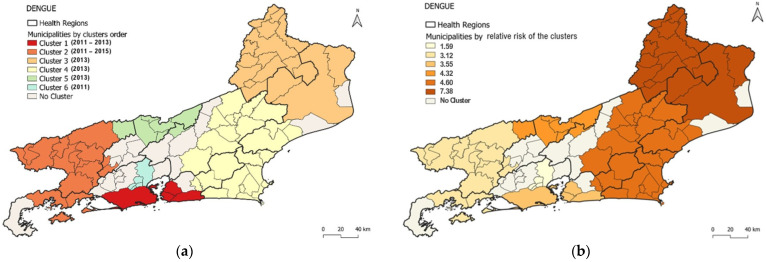

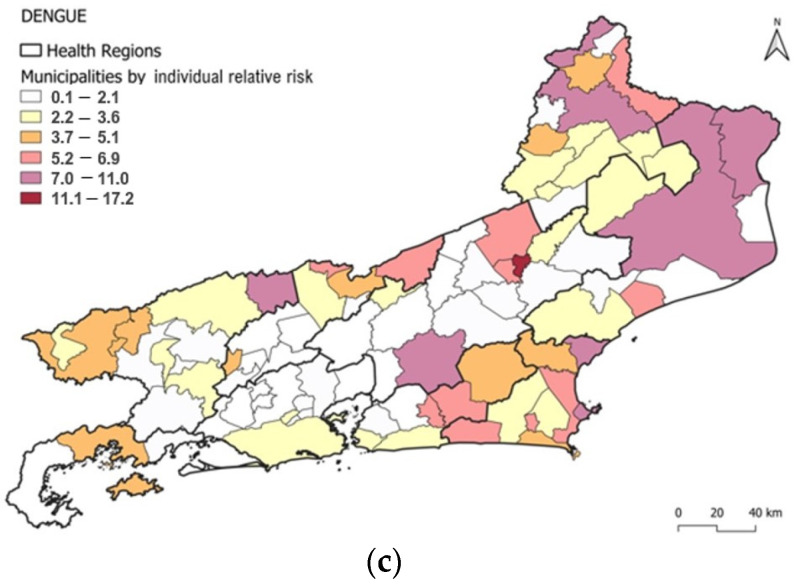
Distribution of dengue clusters according to (**a**) order of most probable clusters, (**b**) relative risk of clusters and (**c**) relative risk of clusters by municipality.

The detected dengue clusters covered 73 of the 92 municipalities in the state (79.3%), with a median of 11 municipalities per cluster. The most likely dengue cluster detected comprised four municipalities distributed in Metropolitan I region, Rio de Janeiro City (the state capital), and Metropolitan II region, Niterói, São Gonçalo, and Maricá (Figure 3a). The cluster was detected from 2011 to 2013 with 395,331 cases in a population of 8,165,684 (annual incidence rate of 1639.5 cases per 100,000 inhabitants). The RR for the cluster was 3.55, with individualized values per municipality ranging from 1.96 (São Gonçalo) to 3.43 (Rio de Janeiro/Capital) (Table 1, Figure 3a,c). The population density of the municipalities included in the primary cluster ranged from 351.55 to 5265.82 (median 3838.35) inhabitants per km^2^ (Figure 1c and Figure 3a). From 2011 to 2013, the HI values for *Ae. aegypti* ranged from 0.6 to 2.0 (2011), 0.3 to 1.6 (2012) and 0.1 to 1.2 (2013).

The cluster with the highest RR (7.38) was the third most likely and comprised municipalities from the northwest and north regions, concentrated solely in 2013 (Table 1 and Figure 3a,b), indicating regions with a higher incidence rate than others in the state.

However, within each cluster, there were municipalities that stood out from others as being distinct. Thus, in the most likely cluster, Rio de Janeiro/Capital (RR: 3.4), Niterói (RR: 2.7), and Maricá (RR: 2.8) had higher RRs than São Gonçalo (RR: 1.9) (Figure 3a,c).

Five additional spatio-temporal clusters of dengue were detected using the model: Cluster 2 (consisting of 15 municipalities) occurred between 2011 and 2015; Clusters 3 (consisting of 17 municipalities), 4 (consisting of 25 municipalities), and 5 (consisting of 7 municipalities) were detected only in 2013; and Cluster 6 (consisting of 5 municipalities) was detected only in 2011. The annual incidence rate observed in these clusters varied from 1001.5 cases (Cluster 6) to 4525.3 cases per 100,000 inhabitants (Cluster 3). Cluster 3 had the highest RR and annual incidence (Table 1). 

In Cluster 2, the HI values for *Ae. aegypti* ranged from 0 to 2 in the 15 municipalities that comprised this cluster from 2011 to 2015. The values ranged from 0 to 2.6 in Cluster 3 (17 municipalities only in 2013), 0 to 1.8 in Cluster 4 (25 municipalities only in 2013), 0 to 1.7 in Cluster 5 (7 municipalities only in 2013); and 0.9 to 2.6 in Cluster 6 (5 municipalities only in 2011).

### 3.3. Spatio-Temporal Cluster Analysis of Chikungunya

**Figure 4:**
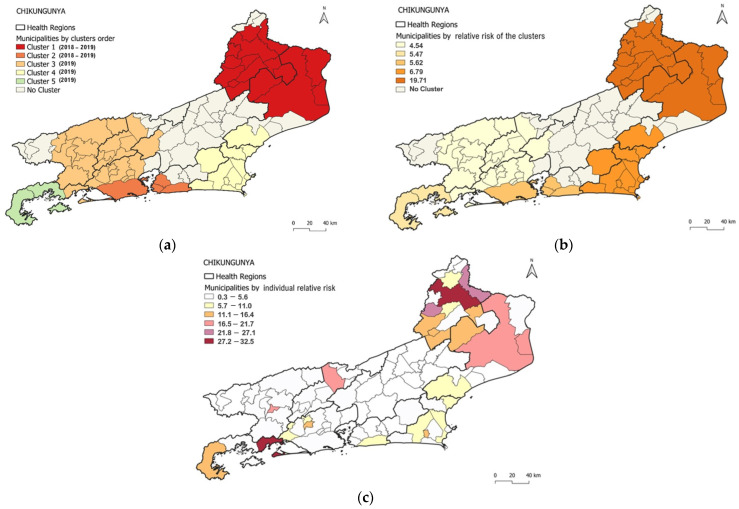
Distribution of chikungunya clusters according to (**a**) order of the most likely clusters, (**b**) relative risk of clusters and (**c**) individual relative risk of clusters by municipality.

The detected chikungunya clusters covered 61 of the 92 municipalities in the state (66.3%), with a median of 11 municipalities per cluster. The most likely chikungunya cluster detected consisted of 16 municipalities: northwest region: Cardoso Moreira, Italva, Cambuci, São José de Uba, Bom Jesus do Itabapoana, Itaperuna, Itaocara, Aperibé, Santo Antônio de Pádua, Miracema, Laje do Muriaé, Natividade; north region: São João da Barra, São Fidélis, Campos dos Goytacazes, São Francisco de Itabapoana (Figure 4a).

The cluster was detected from 2018 to 2019, with 30,728 cases in a population of 906,082 (annual incidence rate: 1637.6 cases per 100,000 inhabitants). The RR for the cluster was 19.71, with values per municipality ranging from 1.25 (Cambuci) to 32.48 (Itaperuna) (Table 2, Figure 4a,c). Population density of municipalities included in the primary cluster ranged from 24.02 to 115.16 (median: 50.475) inhabitants per km^2^. From 2018 to 2019, population density ranged from 24,0 to 115,2 (median: 50.48) inhabitants per km^2^ (Figure 1c and Figure 4a). From 2018 to 2019, the HI values for *Ae. aegypti* ranged from 0 to 4.7 (2018) and from 0.4 to 4.4 (2019).

The most prominent cluster was also the one with the highest RR (19.7) and the highest annual incidence (1637.6) (Table 2 and Figure 4a,b). However, within each cluster, some municipalities stood out, as in the case of the most probable cluster, where Itaperuna (32.5), Bom Jesus do Itabapoana (24.9), and Miracema (22.3) had the highest RR (Figure 4a,c).

Additionally, four spatio-temporal clusters of chikungunya were detected by the model. Cluster 2 occurred from 2018 to 2019 and included four municipalities; in the same way, the most probable dengue cluster comprised Rio de Janeiro/Capital (Metropolitan I region) and Niterói, São Gonçalo, and Maricá (Metropolitan II region). Clusters 3, 4, and 5 were detected only in 2019 and comprised 28, 11, and 2 municipalities, respectively. The annual incidence rate observed in these clusters ranged from 384.7 cases to 657.9 cases per 100,000 inhabitants (in Clusters 2 and 4, respectively) (Table 2, Figure 4). The most likely chikungunya cluster (Cluster 1) and the highest RR cluster were detected in the northwest and north regions of the state, respectively, followed by the municipalities in Metropolitan I and Metropolitan II regions (Cluster 2) (Figure 4a).

In Cluster 2, the HI values for *Ae. aegypti* ranged from 0.4 to 1.7 across 2018–2019 in the 16 municipalities that compose the cluster. The values ranged from 0 to 4.7 in Cluster 3 (four municipalities in 2018–2019); 0.3 to 3 in Cluster 4 (28 municipalities only in 2019); and from 1.1 to 1.8 in Cluster 5 (2 municipalities only in 2019).

### 3.4. Spatio-Temporal Cluster Analysis of Zika

**Figure 5:**
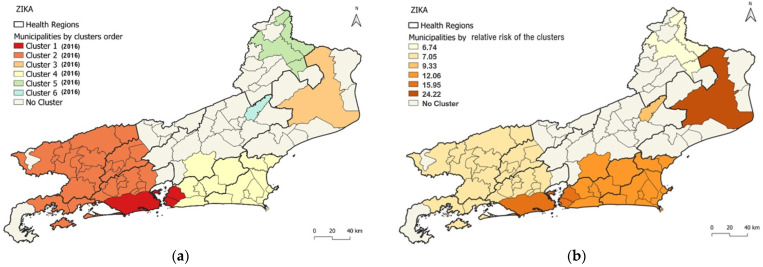

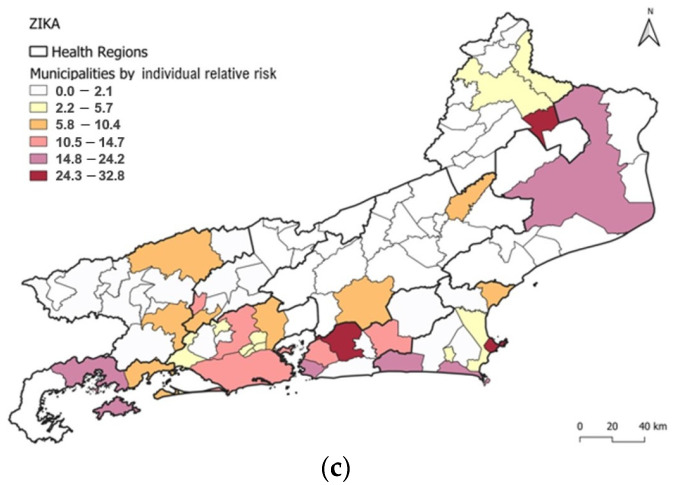
Distribution of Zika clusters according to (**a**) order of the most likely cluster, (**b**) relative risk of clusters, and (**c**) individual relative risk of clusters by municipality.

The detected Zika clusters covered 53 of the 92 municipalities in the state (57.6%), with a median of 4 municipalities per cluster. The most likely cluster (Cluster 1) of Zika comprised three municipalities similar to the most likely clusters of dengue (Cluster 1—Dengue) and the second most likely cluster of chikungunya (Cluster 2—Chikungunya). The municipalities were located in the Metropolitan II region (Niterói and São Gonçalo) and in the Metropolitan I region (Rio de Janeiro/Capital) (Figure 5a). This cluster was detected in 2016 with 44,718 cases in a population of 8,021,163 (annual incidence rate of 555.0 cases per 100,000 inhabitants). The RR of the cluster was 15.95, with values per municipality ranging from 11.62 in Rio de Janeiro/Capital to 18.96 in Niterói (Table 3, Figure 5a,c). The population density of the municipalities included in this cluster ranged from 3640.8 to 5265.82 (median = 4035.9) inhabitants per km^2^ (Figure 1c and Figure 5a). The HI values for *Ae. aegypti* ranged from 0.8 to 1.5 in 2016.

Notably, the cluster displaying the highest RR (24.2) was the third most probable cluster, and it had the highest annual incidence (1362.6) encompassing a single municipality, Campos dos Goytacazes, located in the north region and displaying agglomeration only in 2016 (Table 3 and Figure 5a,b). 

Furthermore, within each cluster, certain municipalities had a higher RR than others. For instance, in the most likely cluster, Niterói displayed an RR of 18.9, which was higher than that for São Gonçalo (12.3) and Rio de Janeiro/Capital (11.6) (Figure 5a,c). In addition, the model identified five other spatio-temporal clusters. Cluster 2 occurred in 2016 and comprised 29 municipalities, while Clusters 3, 4, and 5 emerged in 2016 and comprised 1, 15, and 4 municipalities, respectively. Finally, Cluster 6, detected in 2016, consists of a solitary municipality. The annual incidence rate observed in these clusters ranged from 361.8 cases (Cluster 2) to 1362.6 cases per 100,000 inhabitants (Cluster 3).

The most likely cluster formation for Zika (Cluster 1) was noted in the Metropolitan I and Metropolitan II regions of the state, followed by the second most likely cluster comprising municipalities from Metropolitan I, Middle Paraíba, South Central Fluminense and Ilha Grande Bay regions (Cluster 2) (Figure 5a).

Al six clusters occurred in the year of 2016. HI for *Ae. aegypti* in Cluster 2 ranged from 0 to 1.7 (three municipalities), 1.4 for Cluster 3, 0.1 to 2.8 for Cluster 4 (15 municipalities), 0.3 to 1.2 for Cluster 5 (4 municipalities) and 0 for Cluster 6.

## 4. Discussion

Our investigation of dengue, chikungunya, and Zika cases utilized a spatio-temporal analysis to evaluate the extent to which these arboviruses correlate in time and space, with the goal of identifying clusters with higher risks areas in the Rio de Janeiro state. The analyses showed that different clusters were present for each arbovirus, with varying incidence rates and geographic and temporal extent. We identified specific clusters most likely at higher relative risk. The COVID-19 pandemic had an impact on the management and occurrence of several diseases, including factors associated with risk such as contact rate between humans and vectors of these arboviruses. During the COVID-19 pandemic, there was a significant decrease in reported cases, especially in dengue [7]. Given the pandemic across this study area, arbovirus surveillance and control measures were scaled down due to reallocation of resources to COVID-19 patients and in order to alleviate the strain on healthcare systems. Therefore, it is plausible that arbovirus cases may have been underreported during this time, which could influence our models. To address this issue, the present study limited the analysis until the year 2019.

To accomplish this, the time series data from 2010 to 2019 were analyzed, revealing a pattern of dengue outbreaks with three consecutive epidemics in 2011, 2012, and 2013, which were of greater intensity than the two consecutive epidemics in 2015 and 2016 [5,6]. It is likely that the circulation of chikungunya and Zika during 2014 and 2015 had an impact on the magnitude of dengue epidemics in the state. In fact, during the period of 2015–2019, the Rio de Janeiro state had a complex scenario with the simultaneous circulation of dengue, chikungunya, Zika, and Yellow Fever [5,6,27,28,29]. It is noteworthy that the period from 2010 to 2019 witnessed a significant number of dengue epidemics in the state. This trend is similar to that in the observations in the Americas, where a new outbreak occurred during the period of 2011–2017, coinciding with the large movement of people caused by four global sporting events, the 2011 Pan American Games in Guadalajara (Mexico), the 2013 Confederations Cup (State of Rio de Janeiro, Brazil), the FIFA 2014 World Cup (the state of Rio de Janeiro and others in Brazil) and the 2016 Olympics (the state of Rio de Janeiro, Brazil) [30].

Our results revealed similarities between dengue and Zika in terms of population and population density within their higher-risk clusters. These clusters comprised areas with populations exceeding 8 million inhabitants living in high-risk areas and a median population density of over 4000 inhabitants. Despite the largest populations, Zika exhibited the lowest annual incidence (555.0 cases per 100,000 inhabitants). Conversely, dengue had the highest incidence (1639.5 cases per 100 thousand inhabitants). Additionally, both diseases presented similarities in terms of municipalities within the high-risk areas, predominantly concentrated in the Metropolitan regions of the state, namely Rio de Janeiro/Capital, Niterói, and São Gonçalo. These findings indicate that these few municipalities represented the highest risk areas for dengue and Zika in the state.

Chikungunya only caused an epidemic in 2019 [6] despite imported cases in 2014 and autochthonous cases in 2015. The years 2018 and 2019 represented the most probable cluster for chikungunya, exhibiting a distinct pattern compared to dengue and Zika. Within this cluster (Cluster 1), several municipalities in the northwest and north regions were identified as having the highest RR and annual incidence. For dengue and chikungunya, the highest relative risk (RR) and annual incidence were not observed in Cluster 1, but rather in Cluster 3, indicating another similarity between dengue and Zika. 

Chikungunya and dengue had equal medians for the number of municipalities per cluster (11 municipalities), and the number of clusters spanning more than one year. Unlike Zika, which had a median of four municipalities per cluster, chikungunya and dengue had a different profile, indicating persistence over a longer period. The occurrence profile of Zika was rapid and more concentrated, reaching transmission peaks in a shorter time and covering smaller geographic areas. This may suggest a higher percentage of asymptomatic individuals, a faster depletion of the susceptible population, or a more efficient infection rate for Zika.

Although the spatio-temporal distribution of each arbovirus in the state varied in terms of area and time, there was commonality in the persistence of risk areas for the three circulating arboviruses. The municipalities of Niterói and São Gonçalo in the Metropolitan II region along with Rio de Janeiro/Capital in the Metropolitan I region were predominant in the most likely clusters identified. Dengue Cluster 1, chikungunya Cluster 2 and Zika Cluster 1 exhibited similar patterns, encompassing Niterói and São Gonçalo in Metropolitan II region and the Municipality of Rio de Janeiro/Capital in the Metropolitan I region. This highlights the importance of these metropolitan cores in maintaining the transmission of arboviruses. Aiming to classify dengue transmission profiles, a study in Brazil during the period from 2010 to 2019 categorized most of the municipalities of the state of Rio de Janeiro as exhibiting an epidemic and persistent transmission profile. Municipalities with higher incidence and larger populations tend to be classified as having persistent transmission, suggesting the existence of critical community sizes [31]. 

The SaTScan software successfully identified clusters of arbovirus infections, showing risk heterogeneity at a local level, as previously described for other mosquito-borne diseases such as malaria [32]. In addition, our findings underscore the importance of using methods and tools such as Scan analysis and interactive dashboards, respectively, for arboviruses surveillance, providing a better understanding of the spatio-temporal dynamics of dengue, chikungunya, and Zika in different municipalities of the state. In response, we developed an arbovirus dashboard that includes an epidemiologic profile and a dengue control chart, following the methodology recommended by the Ministry of Health [33]. This allowed us to outline the curve of the expected number of cases by the epidemiological week of symptom onset and the maximum limit defining and visualizing disease occurrence throughout the year, health regions, and municipalities. The tool was made available to be used by the Health Surveillance service of SES/RJ. It was published in 2022 on the secretariat’s website, in the arbovirus’s dashboard, which contributes to the characterization of endemic and epidemic episodes in the Rio de Janeiro state [34]. 

In a previous study [6], we identified the years 2015 and 2016 as the years of epidemic for dengue based on an annual incidence cutoff of 300 cases per 100,000 inhabitants. In this study, the years 2011–2013 appeared in the first high-risk cluster, and 2015 appeared in the second high-risk cluster, reinforcing the previous findings. However, the year 2016 did not appear in any of the high-risk dengue clusters, suggesting that despite having an incidence of above 300, it may not have been a high-risk year for dengue. Zika, on the contrary, was the only arbovirus with a high-risk year in 2016. 

In our previous findings [6], challenges were faced in clearly defining the priority regions using the annual incidence of 300 as a cutoff point for higher-risk areas. Therefore, only the northwest region was highlighted in 2016 as a high-risk area for dengue; the northwest region was also highlighted for chikungunya in 2019 and the Metropolitan II and Ilha Grande Bay regions were highlighted for Zika. However, in the present study, the high-risk regions for each of these arboviruses were clearly defined. Notably, dengue exhibited the highest persistence of risk areas in the state. For the three arboviruses, the regions with the highest population densities, as well as those with the smallest populations, were at higher relative risk and deserve concentration of control and prevention actions. Collectively, the findings between the studies are complementary, emphasizing the importance of epidemiological monitoring indicators and tools such as case incidence rate, time curves of cases and control diagrams. These tools can be used in conjunction with more robust analyses within health services.

In summary, the most likely cluster of dengue and Zika cases is in certain municipalities in the Metropolitans regions of the state (Rio de Janeiro/Capital, Niterói and São Gonçalo). For chikungunya, the most likely cluster encompasses several municipalities in the northwest and north regions (Cardoso Moreira, Italva, Cambuci, São José de Uba, Bom Jesus do Itabapoana, Itaperuna, Itaocara, Aperibé, Santo Antônio de Pádua, Miracema, Laje do Muriaé, Natividade; São João da Barra, São Fidélis, Campos dos Goytacazes, and São Francisco de Itabapoana). However, when considering the highest relative risk of clusters, individuals residing in the northwest and north regions faced a greater risk of dengue and chikungunya compared to those outside these regions. Specifically, Campos dos Goytacazes in the north region stands alone as the cluster with the highest relative risk for Zika, while the first most likely cluster has the second highest relative risk. Dengue presented most likely clusters persisting for up to 5 years (2011 to 2015), chikungunya had most likely clusters in the years 2018 and 2019 and Zika only presented most likely clusters in 2016.

Therefore, this study emphasizes the importance of analyzing individual municipalities within clusters to obtain a more nuanced understanding of the spatial distribution of notified cases of dengue, chikungunya, and Zika. Moreover, a heterogeneous pattern was observed in the significant clusters detected for each arbovirus. Our data reinforce the notion that the identification of high-risk clusters of dengue, chikungunya, and Zika, and the differences in the pattern of profile between these arboviral diseases in space and time enable policy efforts to be focused more effectively on prevention and control of these urban arboviruses.

## Figures and Tables

**Table 1:** Most likely cluster analysis of dengue in Rio de Janeiro state, 2010–2019.

Cluster	No. of Locations	Period	No. of Cases	Population	Annual Incidence Rate ^a^	Relative Risk	LLR	*P*
1	4	2011–2013	395,331	8,165,684	1639.5	3.55	169,431.528	<0.001
2	15	2011–2015	105,720	1,165,671	1841.1	3.12	45,829.719	<0.001
3	17	2013	40,285	900,056	4525.3	7.38	45,121.201	<0.001
4	25	2013	41,183	1,495,223	2824.7	4.6	30,093.344	<0.001
5	7	2013	5,114	189,491	2725.7	4.32	3541.912	<0.001
6	5	2011	21,211	2,162,210	1001.5	1.59	1945.04	<0.001

^a^ per 100,000 population. Abbreviation: LLR, log-likelihood ratio.

**Table 2:** Likely cluster analysis of chikungunya in Rio de Janeiro state, 2015–2019.

Cluster	No. of Locations	Period	No. of Cases	Population	Annual Incidence Rate ^a^	Relative Risk	LLR	*P*
1	16	2018–2019	30,728	906,082	1637.6	19.71	59,534.31	<0.001
2	4	2018–2019	64,933	8,165,684	384.7	5.62	47,743.47	<0.001
3	28	2019	20,022	4,668,653	414.7	4.54	13,908.13	<0.001
4	11	2019	7336	1,013,163	657.9	6.79	7672.22	<0.001
5	2	2019	1348	226,745	547.3	5.47	1185.31	<0.001

^a^ per 100,000 population. Abbreviation: LLR, log-likelihood ratio.

**Table 3:** Most likely clusters of Zika in Rio de Janeiro state, 2015–2019.

Cluster	No. of Locations	Period	No. of Cases	Population	Annual Incidence Rate ^a^	Relative Risk	LLR	*P*
1	3	2016	44,718	8,021,163	555	15.95	69,429.54	<0.001
2	29	2016	16,940	4,656,627	361.8	7.05	17,363.67	<0.001
3	1	2016	6652	483,435	1362.6	24.22	14,608.92	<0.001
4	15	2016	8895	1,300,690	665.6	12.06	13,635.88	<0.001
5	4	2016	649	159,871	403.1	6.74	684.2	<0.001
6	1	2016	51	9076	560.8	9.33	68.36	<0.001

^a^ per 100,000 population. Abbreviation: LLR, log-likelihood ratio.

## Data Availability

Not applicable.
